# Dispensing Anomalies in Public Institutions

**Published:** 1912-08-31

**Authors:** 


### DISPENSING ANOMALIES IN PUBLIC INSTITUTIONS.

### The Need for a Uniform System.

The pharmacist from a foreign country, when he hears for the first time of the strange anomalies of the law relating to the sale of poisons and the dispensing of medicines in force in England, never fails to be very much amused. Our own legislators, if they gave just a few moments consideration to the matter, would be amazed. As a matter of fact there are very few members of Parliament who have deemed it worth their while to make anything like a proper study of the strangely intricate and yet hopelessly insufficient legislation affecting the distribution of medicines to the British people; and even Cabinet Ministers belonging to both political parties have been known to say things which showed very clearly that their knowledge of what is popularly called the poison law is in its infancy.

This legislation, however, is an excellent example of a condition which theoretically ceased to exist long ago—namely, "one law for the rich, another for the poor." It would be nearer the truth to write it, "one law for the rich, none for the poor," for while, as a rule, the rich man need have no fear that his medicines will be dispensed under improper conditions, the poor man can seldom be sure that his will be compounded either by qualified or experienced persons or under the regulations which govern the compounding of drugs in properly ordered places. It is in fact lawful for any person, irrespective of age, experience or qualification, to dispense medicines; on the other hand, it is unlawful for anyone except a duly registered chemist and druggist to sell by retail a medicine containing a poison. The critical act is the selling not the compounding, and as the distribution of medicines to hospital patients does not involve a sale there is nothing in the Pharmacy Acts to require that the dispensing in hospitals and other public institutions shall be done by qualified persons or that the poisons at these institutions shall be stored in special bottles or kept apart from non-poisonous substances, or that poisonous liniments and lotions shall be handed over to patients in bottles distinguishable by touch from ordinary bottles. Fortunately, however, that part of the public which depends for its medicine on charitable institutions does not often suffer as a result of the insufficiency of the law, for in actual practice all desirable precautions are taken to ensure a skilful and safe service. In all the great London hospitals the dispensing staff consists solely of duly registered pharmacists and in the larger provincial institutions the chief dispenser at least is a qualified pharmacist.

Similarly the qualification of the Pharmaceutical Society is held by the dispenser in each of the mental hospitals under the control of the London County Council in which a dispenser is employed, and the appointment of Poor-Law dispensers is subject to the approval of the Local Government Board, whose regulations require the person appointed to possess one of the following qualifications: (1) registration under the Pharmacy Act, 1868; (2) registration as a pharmaceutical chemist in Ireland; (3) the licence of the Society of Apothecaries; (4) the Assistant's Certificate of the Society of Apothecaries; (5) registration as an Army compounder, R.A.M.C. The dispensers at the principal naval stations must possess the qualification of the Pharmaceutical Society of Great Britain or of Ireland, but at other naval hospitals and dispensaries persons who do not possess either of these qualifications are employed. Candidates for appointments as dispensers in H.M. prisons must hold the Pharmaceutical Society's diploma, but for posts under the Metropolitan Asylums Board holders of the Assistant's Certificate of the Society of Apothecaries are eligible as well.

Some years ago the Council of the Pharmaceutical Society appointed a special committee to inquire into the qualifications of dispensers in public institutions; the committee obtained particulars of 694 dispensaries, and found that in 381 cases dispensers were appointed, while in 313 cases there were no regular dispensers. Of the 381 institutions where dispensers were appointed, persons registered under the Pharmacy Act were employed in 214 instances, apothecaries' assistants in 37, Army compounders in 20, while in 110 cases the dispensers held no qualification. Of the 313 cases in which regular dispensers were not appointed it was found that in 211 institutions the dispensing was performed under medical responsibility, and in 102 cases by local chemists, etc. In fact, in 527 of the 694 dispensaries concerning which particulars were obtained—75.9 per cent.—the responsibility for the dispensing of medicines was in competent hands. The impression the committee obtained from these figures was that, as a general rule, public authorities conducting or controlling hospitals, infirmaries, or dispensaries are fully aware of the importance of the work of compounding and dispensing medicines, and are careful to protect the patients in such institutions by appointing dispensers who possess a statutory qualification; but that in the case of a number of voluntarily supported institutions, with possibly the most urgent reasons for exercising a rigid economy, the protection of patients from unqualified dispensers was not a primary consideration.

Although it is the practice of the authorities responsible for the conduct of the large hospitals and of various State and public departments to insist upon the employment of adequately qualified persons as dispensers, there is nothing in law to compel them to do so, and it is not the invariable practice of smaller institutions to follow the custom of the large hospitals. The adoption of some uniform system throughout the United Kingdom seems desirable, but until some alteration of the Pharmacy Acts is made this is hardly possible. The dispensing of medicines in public institutions is an extremely important service, and there is no logical reason why the safety of a patient in a small provincial hospital should not be ensured by the same conditions as in the large institutions of London.

